# Butylated hydroxytoluene protects bull sperm surface protein-P25b in different extenders following cryopreservation

**DOI:** 10.14202/vetworld.2020.649-654

**Published:** 2020-04-11

**Authors:** A. M. Khumran, N. Yimer, Y. Rosnina, H. Wahid, M. O. Ariff, H. Homayoun, K. Asmatullah, T. K. Bello

**Affiliations:** 1 Department of Theriogenology and Production, Faculty of Veterinary Medicine, Ahmadu Bello University, Zaria, Nigeria; 2 Department of Veterinary Clinical Studies, Faculty of Veterinary Medicine, Universiti Putra Malaysia, 43400 UPM Serdang, Selangor, Malaysia; 3 Department of Veterinary Pre-clinical Studies, Faculty of Veterinary Medicine, Universiti Putra Malaysia, 43400 UPM Serdang, Selangor, Malaysia; 4 Department of Veterinary Pathology and Microbiology, Faculty of Veterinary Medicine, Universiti Putra Malaysia, 43400 UPM Serdang, Selangor, Malaysia; 5 Biotechnology Research Programme, National Animal Production Research Institute, Zaria, Nigeria

**Keywords:** bull sperm, butylated hydroxytoluene, extender, P25b, Western blotting

## Abstract

**Aim:**

The aim of this study was to investigate the effects of different concentration of butylated hydroxytoluene (BHT) on sperm membrane surface protein “P25b” from cryopreserved bull semen in either lecithin based Bioxcell^®^ (BX) or two egg-yolk based extenders, tris-egg yolk (TEY), and citrate-egg yolk (CEY).

**Materials and Methods:**

Forty-five semen samples, 15 each were extended with either BX, TEY, or CEY extender which contained different concentrations (0.0 - control, 0.5, 1.0, 1.5, 2.0, and 3.0 mM/mL) of BHT. The extended semen samples were frozen at a concentration of 20×10^6^/mL in 0.25 mL straws and stored in liquid nitrogen for 2weeks. The frozen samples were thereafter thawed, proteins extracted and analyzed for quantities of protein P25b through direct sodium dodecyl sulfate-polyacrylamide gel electrophoresis gel densitometry. Peptides were confirmed by Western blotting (WB).

**Results:**

Results showed that supplementation of BHT improved (p<0.05) quantity of protein P25b at concentrations of 0.5mM/mL for BX and at 1.0 mM/mL for TEY and CE when compared with the controls and other treatments.

**Conclusion:**

BHT supplementation at 0.5 in BX and 1.0 mM/mL in TEY and CEY has protected bull sperm fertility marker protein P25b in frozen-thawed bull sperm.

## Introduction

Semen cryopreservation has allowed specific opportunities for the conservation of genetic resources through semen banks, guarantee of a constant commercial supply of semen, and collaboration in breed improvement programs by artificial insemination[1]. It has been agreed on, that sperm fertility is challenged following cryopreservation. This occurs due to biophysical damage on spermatozoa that results from the effects of freezing and thawing procedures. The consequences of these procedures are numerous and multifactorial, involving temperature, osmotic, and oxidative effects. During cryopreservation, sperm damage can occur due to damage to the membrane structure due to lipid peroxidation [2]. Cholesterol to phospholipid ratio of sperm bio-membranes gets disturbed mainly due to cholesterol efflux and the generation of numerous reactive oxygen species (ROS)[3]. The resultant sperm cryo-injuries also involved subtle damage that partially explained the reduced sperm fertility after thawing, such as the damage to the bull sperm surface protein “P25b.” Certain proteins, including P25b produced by epithelial cells, are acquired from the lumen of the epididymis by spermatozoa during transit. The intraluminal protein sperm surface interaction anchored the proteins to the sperm membrane and modified the sperm head such that these proteins behaved like an integral part of sperm when submitted to different treatments [4]. This process is essential to generate actively motile and fertile sperm[5], with P25b playing a major role in the regulation of cellular activities that lead to acrosome reaction, recognition, binding, and sperm penetration of zona pellucida of the oocyte during fertilization[6]. Other proteins that are considered homologous to P25b in the bull sperm (such as P34H of the human and P26h of the hamster) have been identified, absence of which has been associated with infertility [7] thus they are considered as fertility markers. Similarly, Lessard *et al*. [7] demonstrated a significant decrease in P25b level after cryopreservation with different magnitude of effect in either egg yolk or milk-based bull semen extender.

Protection of P25b is therefore essential as part of the explanation for reduced bull sperm fertility after cryopreservation. In recent times, the use of antioxidant such as butylated hydroxytoluene (BHT) as a supplement in semen extenders has been shown to improve semen quality parameters such as motility and viability, which is thought to have resulted from its control of oxidants such as ROS. BHT acts as an antioxidant preservative, antiviral, and antimicrobial agent. BHT is an organic soluble molecule, used to stop the auto-oxidation of lipid bilayer and membrane of sperm cells[8]. BHT also scavenges ROS from the surroundings of the spermatozoa and converts these molecules into hydroperoxides [9], thereby reducing the harmful effects of ROS’ on sperm cells during storage. Its use efficiently inhibits lipid peroxidation reactions in biological membranes. The BHT supplementation of semen extender has improved sperm quality and reduced lipid peroxidation in different species [10]. In this light, some bull sperm parameters and damage to DNA and acrosome were protected by BHT antioxidant treatments in both chilled and frozen-thawed bull sperm Khumran *et al*. [11] and Mostafa *et al*. [12].

The aim of the present study was to investigate the effects of different concentration of BHT on sperm membrane surface protein P25b from cryopreserved bull semen in either lecithin (Bioxcell^®^ [BX]) or two egg yolk (tris-egg yolk [TEY] and citrate-egg yolk [CEY]) based extenders. The present study was designed to test the hypothesis whether appropriate levels of BHT concentration supplementation will exert a positive protective effect on surface protein P25b of post-thawed bull semen.

## Materials and Methods

### Ethical approval

The study was conducted in accordance with the guideline of the Institutional Animal Care and Use Committee (IACUC) of Universiti Putra Malaysia, with AUP No: R073/2015.

### Study design, study period, animals, and management

The study was conducted between 2015 and 2016 at the Theriogenology and Cytogenetics Unit, Department of Veterinary Clinical Studies, Faculty of Veterinary Medicine, Universiti Putra Malaysia, Serdang, Selangor. Semen samples were collected from bulls at the Taman Pertanian Universiti farm. Protein was extracted, demonstrated, and estimated from bulls’ semen samples extended with either of three semen extenders at varying concentrations of BHT, which was kept for 14days. Three crossbred bulls; Simmental×Brangus, Brangus×Hereford, and Kedah-Kelantan×Brangus were used for semen collection. The bulls were apparently healthy, sexually mature, and fertile. The average age and body weight of the bulls were 5.3±0.3years old and 649.3±9.7kg, respectively. They were maintained under uniform management conditions, fed with freshly harvested Surinam grass (*Brachiaria decumbens*), supplemented with concentrate, palm kernel cake, and provided with mineral licks and water *ad libitum*.

### BHT dose determination

The BHT used this study was purchased from Sigma-Aldrich-catalog# B1378-500G (molarity = 220). One hundred mM/mL stock solution was obtained by dissolving 220g of the BHT white crystals in 1L of absolute ethanol (99%). The stock was then used subsequently to obtain the desired concentration for every treatment. For example, 10 µL of the stock already contains 1 mM of BHT was dissolved in 2mL of a diluted semen sample to obtain 0.5 mM/mL of BHT concentration. However, the stock solution and diluted sample had not been directly mixed together because the ethanol-containing in the stock solution was detrimental to spermatozoa. Therefore, a measure of the stock had always been put into a clean empty test tube first, warmed, and allowed the ethanol to evaporate leaving the dried BHT crystals to attach to the tube before an appropriate amount of diluted semen sample was added to the test tube which was adequate to make up the desired concentration.

### Samples preparation

Fresh semen samples that had more than 80% morphologically normal sperm, more than 70% general motility and a concentration of more than 500×10^6^ sperm/mL were extended with either BX, TEY, or CEY extenders supplemented with BHT concentrations of 0 (control), 0.5, 1.0, 1.5, 2.0, and 3.0 mM/mL. The extended samples were left in a water bath at 37°C for 5min to allow uptake of BHT by spermatozoa. Next, the samples were frozen, according to Khumran *et al*.[11] in liquid nitrogen and stored for 14days.

### Protein extraction and estimation

The frozen semen samples containing various BHT concentrations and control were thawed in a water bath at 37°C for 30 s. Four straws of thawed semen were randomly pooled together from each treatment group and control in separate test tubes. Live and motile spermatozoa were then separated from the dead by the swim-up procedure. Following swim-up separation through sperm-TALP medium, the dead spermatozoa were decanted, and the live ones were further used to extract proteins according to the procedure described by Lessard *et al*. [7]. First, the spermatozoa were washed 2times with Dulbecco’s phosphate-buffered saline (D-PBS), lysed in lysis buffer (0.2% Triton×100 in D-PBS), pelleted, and finally, proteins were precipitated overnight in ice-cold acetone and then re-suspended in sample buffer. Extracted proteins in sample buffer were treated with protease inhibitor (mix) at 10 µL/mL to protect proteins from denaturation and kept at −20°C until use. All samples were prepared in duplicates: One member was used for estimation of total protein and the other for sodium dodecyl sulfate-polyacrylamide gel electrophoresis (SDS-PAGE) and subsequently either densitometry or Western blotting (WB) procedures to determine protein P25b.

The amount of proteins in each sample per microliter was estimated by a modified Bradford procedure described by Sarsaifi *et al*. [13]. One mL Bradford reagent was mixed with 100 µL samples and bovine serum albumin standards (0, 20, 40, 60, 80, and 100%). The reaction absorption was read in a spectrophotometric ELISA reader, equipped with Magellan (7.1 sp1) software (Sunrise. TECAN, Australia). The mixture was loaded into a 96 micro well-plate, first incubated for 5min at room temperature (26°C) then the absorbance was read automatically by the machine at 750 MHz (wavelength). Results obtained from the samples were compared with the standard curve equation. “Y” was determined as the amount of proteins per microliter of a given sample, which was used to load an equal amount of protein onto the gel during SDS-PAGE.

### WB

#### Sample preparation for WB

The frozen protein samples and protein ladder (PL) (GeneDirex^®^, BLUeye Prestained Ladder) were thawed at room temperature (26°C). Each sample was mixed with sample loading buffer at 1:1 ratio, heated at 95°C for 5min before loading into the gel well. Five µL of the ladder were loaded into the first well, followed by 30µg of protein samples (in loading buffer) in the subsequent second to seventh wells and negative control (30 µL of distilled water) in the eighth well.

#### SDS-PAGE

A 12% resolving and 4% stacking gels were prepared, according to Tam *et al*. [14]. First, resolving gel was loaded until the green mark of the electrophoresis gasket apparatus, then stacking gel was used to top up the glass gel cast holder. The casted gel was allowed to stand for 2h for polymerization and became solid. The polymerized gel was then mounted and ran in a Bio-Rad’s (Mini-PROTEAN^®^ Tetra Vertical Electrophoresis) Cell at 80 volt for 2h to separate the proteins based on their molecular weights (MW). Two replicates of gel were prepared: One stained with Coomassie blue staining and the other for immune-blotting, detection, and subsequent P25b expression.

#### Coomassie blue staining and protein P25b densitometry

**Figure-1:**
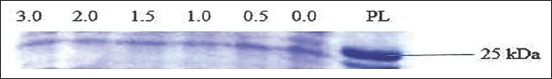
A Coomassie brilliant blue-stained sodium dodecyl sulfate-polyacrylamide gel electrophoresis gel showing bands. PL=Protein Ladder, 0.0=Control. Butylated hydroxytoluene concentrations (0.5-3.0 mM/mL) treatment wells.

Coomassie brilliant blue (CBB) stain was prepared (0.1 % [w/v] CBB R-250, 40% [v/v] methanol, 10% [v/v] acetic acid) in double-distilled water (ddH_2_O) v/v solution [14]. One of the pair of SDS gels was stained for 30min at room temperature (26°C) with agitation, then destained with a destaining solution 10 % (v/v) methanol, 10% (v/v) acetic acid) 2times each time for 15min and the gel was kept overnight the third time in the destaining solution. The separated proteins appeared as blue bands on the gel (Figure-1), according to their MW against the PL. Protein P25b had 25kDa and located at the ladder-green band level.

Protein quantification and analysis were conducted directly from the stained gels in the laboratory. Forty-five CBB-stained SDS-PAGE gels, 15 each from either BX, TEY, or CEY extender, were evaluated by densitometry for quantities of P25b. ImageJ software version IJ 1.46r (a free downloadable image processing software developed at theNational Institutes of Healthand the Laboratory for Optical and Computational Instrumentation (LOCI), University of Wisconsin). Available at https://imagej.nih.gov/ij/download.html) was used for protein quantification, according to Miller [15]. Ascanned gel image was uploaded onto the software; the colored scan was converted to grey at 32 bits and contrast adjusted. Protein bands were then mapped out in rectangular boxes and the selected area-lane-plotted. The plotted lane appears as vertical peaks indicating the intensity of the selected band. The straight line tool was then selected to mark the width of the base of the peaks made by the intensity of the bands. The peak area was selected by the wand tool and later labeled as percentages using the “label peak” option.

#### Protein Immunotransfer and immunoblot

Separated proteins on the second pair of the SDS-PAGE-gel were immuno-transferred to nitrocellulose membrane (BioRad, Hercules, California USA). The protein immuno-transfer was accomplished in a sandwich consisting of the gel, nitrocellulose, and blotter paper (BF3, 145×215 mM. Santa Cruz, USA). The sandwich was first soaked in transfer buffer for 20min before running. Semi dry transfer cell (SD Tans-Blot, Bio-Rad) was used at 25 volts for 2h.

**Figure-2:**
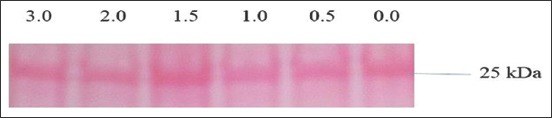
Ponceau-S stained nitrocellulose membrane showing transferred protein bands. 0.0=Control. Butylated hydroxytoluene concentrations (0.5-3.0 mM/mL) treatment wells.

The nitrocellulose carrying the transferred protein was washed in dH_2_O 3times, then incubated in Ponceau-S (PS) stain for 20min to confirm the transfer before proceeding to WB. The red protein bands (Figure-2) on the membrane after the PS-stain indicate quantities of proteins that have been transferred. The gel from which the protein has been transferred was also stained with CBB stain, this would further confirm if the transfer was complete in which case, there would be a plain gel after destaining (without bands) or a partial transfer in which case there would still be remaining bands on the gel after destaining.

The membrane was first stained with PS-stain in order to detect protein bands and ensure complete transfer from the gel, then washed thoroughly with ddH_2_O, then incubated for 1h at room temperature in a blocking buffer. Following blocking, the nitrocellulose was washed 3times, 5min each in Tris-Buffer Saline and Tween 20 (TBS-T) and thereafter, incubated in primary antibody (1°-Ab), Rabbit-polyclonal RAB3B (NBP2-20045, NOVUS BIOLOGICALS) overnight at 4°C at 1:1000 dilution with Ab-dilution solution (5% fat-free skimmed milk in TBS). The 1°-Ab was decanted the following day and the blot was washed in TBS-T as before, then incubated for 1h at room temperature in secondary antibody (2°-Ab), Goat anti-rabbit immunoglobulin G (IgG) antibody horseradish peroxidase conjugate (NB730-H, NOVUS BIOLOGICALS) at 1:30,000 dilution. The washing solution was again used to wash the blotted nitrocellulose 3times as before and the enzyme, TMB membrane peroxidase substrate (KPl) was used to incubate the blotted membrane for 15min. Rabbit polyclonal anti-beta-actin antibody (ab8227, Abcam^®^), known to detect beta-actin proteins at 40kDa MW, was used as a positive control. These antibody reactions were stable in all three extender groups.

### Statistical analysis

Statistical analysis system (SAS V 9.1, SAS Inst. Inc. Cary, North Carolina) was used for analysis. Data were checked for normality using the univariate procedure and the results are presented as means±standard errors of the mean. The relative density (RD) of the protein bands area of each treatment in relation to the control for each extender (BX, TEY, and CEY) was analyzed by one-way ANOVA. Duncan’s multiple range test was used as *post hoc* against control. p<0.05 was considered significant.

## Results

### Protein P25b expression by WB

**Figure-3:**
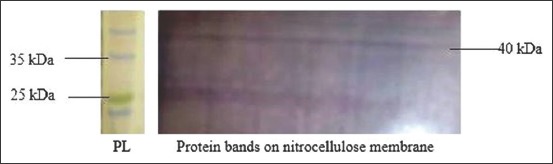
A Western blot showing very faint protein bands, beta-actin reacted at 40 and P25b at 25 kDa.

Antigen-antibody complexes were expressed as dark blue stains on the blotted membrane. P25b protein precipitated at 25kDa MW level as indicated by the BLUeye^®^ PL when used in Tris-glycine-20% buffer. The dark blue stains protein bands were visible as confirmation of the bands on the corresponding Coomassie blue-stained gel (Figure-3).

### Protein densitometry

**Figure-4:**
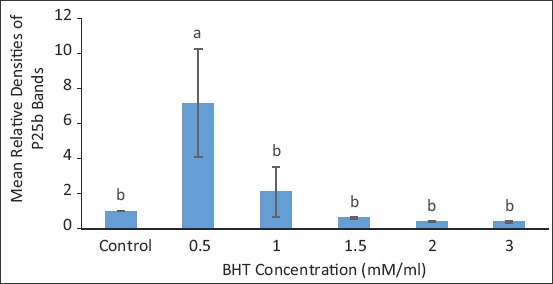
Relative densities of protein bands in treatments compared in Bioxcell^®^ extender. Data are expressed as means ± standard error, n=15. Different letters a, b denote differences (p<0.05) between treatments.

The areas marked by the intensity of the protein bands were measured and resultant RD of the treatments compared to the control for BX extender is shown in Figure-4. The protein was significantly preserved at BHT concentration of 0.5 mM/mL than in the control and treatments at BHT concentrations of 1.0, 1.5, 2.0, and 3.0 mM/mL. The protein was most severely damaged at the BHT concentration of 3.0 mM/mL.

**Figure-5:**
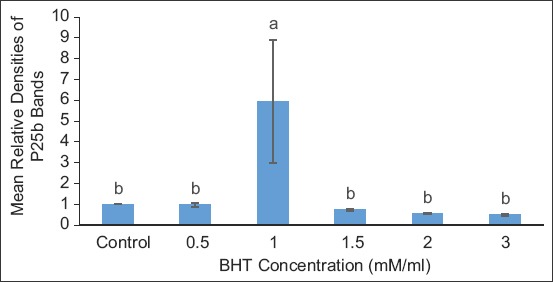
Relative densities of protein bands in treatments compared in tris-egg yolk extender. Data are expressed as means±standard error, n=15. Different letters a, b denote differences (p<0.05) between treatments.

Figure-5 shows the RD of the protein bands in the treatments compared to the control for TEY extender. The intensity of the protein bands was the same in control and treatment concentrations of 0.5, 1.5, 2.0, and 3.0 BHT mM/mL, in which the protein P25b was significantly lower than in the treatment concentration of 1.0 BHT mM/mL.

**Figure-6:**
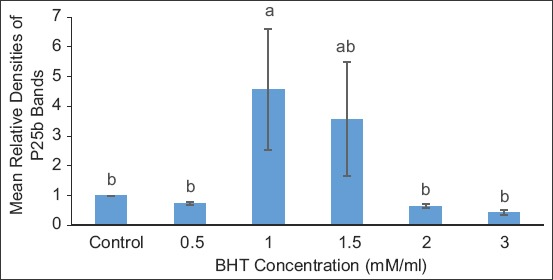
Relative densities of protein bands in treatments compared in citrate-egg yolk extender. Data are expressed as means±standard error, n=15. Different letters a, b denote differences (p<0.05) between treatments.

In Figure-6, the protein P25b was significantly better in BHT concentration of 1.0 mM/mL group than in control, 2.0 and 3.0 mM/mL BHT concentration then followed by 1.5 mM BHT which appeared to be the same with the control, 1.5, 2.0, and 3.0 mM/mL.

## Discussion

The effects of various BHT treatments and controls on bull fertility marker protein P25b were elucidated in this study. The quantification of the protein in question was conducted by direct densitometry from CBB-stained SDS-PAGE gel. To ensure that we were dealing with the target protein of interest, the band corresponding to 25kDa MW was identified, and its specific reactions with primary (Rabbit-polyclonal RAB3B) and secondary antibody (Goat anti-rabbit IgG) complexes were detected by WB. The WB result detected the P25b at 25kDa. This is in tandem with the MW position at which Lessard *etal*.[7] detected the same protein by WB [5]. Detected P25b at 28kDa, but this is not uncommon that protein peptides showed up slightly higher or lower than its theoretical MW position. Although, one particular reason has not been agreed on, many factors (such as incomplete unfolding and modification due to others) are thought to influence the mobility of proteins on gel, thus making the proteins appear slightly bigger or lesser than expected[16].

SDS-PAGE gel densitometry revealed that P25b was protected in optimum treated bull sperm samples than in control, precisely at BHT concentration of 0.5 mM/mL in BX, 1.0 mM/mL in TEY, and 1.0 to 1.5 mM/mL in CEY. By implication, the use of antioxidant BHT at appropriate concentrations in different extenders improved the protection to the bull fertility marker protein during cryopreservation. The BHT concentration that gave optimum protection in this study was similar to report by Mostafa *et al*. [12] using BHT to preserve buffalo semen. This is understood to influence the fertility potential of frozen-thawed bull spermatozoa, since this protein partakes in the process of fertilization and that its low abundance or absence was partly responsible for infertility of some bulls [17]. It has also been reported that the protein P25b concentration depleted significantly after cryopreservation resulting in diminished fertility [7]. The findings in the current study are in agreement with Karunakaran and Devanathan [18] in which they reported the abundance of fertility-related proteins such as P25b in fertile bulls and the proteins’ absence in the infertile bull. This implies that if fertility is reduced due to either low levels or damage of P25b by cryopreservation, the fertility could be saved by protecting such protein. The outcome in this study further reiterates the superiority of BHT’s treatment at those optimum concentrations for the different extenders. In recent studies, Khumran *et al*. [11] demonstrated that BHT had improved the quality parameters (general motility, progressive motility, morphology, acrosome integrity, DNA integrity, and malondialdehyde of sperm) of the bull semen when added at the same concentrations of 0.5 in BX and 1.0 to 1.5 mM/mL in TEY and CEY as it is the case in the present study. However, it is also observed that, unlike in the previous studies where some bull sperm[19], buffalo sperm [12] quality parameter characteristics deteriorated significantly against the control in higher BHT treatment groups. Here, the RD of protein in BHT treatment groups (other than the optimum groups of 0.5 in BX, 1.0 in TEY and CEY) remained the same with the control in all extenders. This may mean that, though the control might be better than other treatment in certain parameters, such was not adequate to produce the difference in abundance of the fertility marker protein between them.

Although the WB result detected the protein antibody reactions, this only confirmed the identity of protein that we were dealing with. However, the observed bands were very faint even in the samples with the optimum treatment in all the extenders. The same was reported in the previous studies where P25b was expressed. The protein P25b protein bands were more robust on SDS-PAGE gel than on membranes after WB in this study and also in Lessard *et al*. [7] and Parent *et al*. [17]. This is believed to happen because the processes involved in WB usually expose the protein to various factors that could lead to either partial loss of protein or dark membrane backgrounds that may camouflage with the bands, making them barely visible [20,21].

## Conclusion

Bull sperm surface protein P25b was protected during cryopreservation when BHT was used at 0.5 mM/mL in BX and 1.0 mM/mL in TEY and CEY extenders. However, the damage to this protein was not significant between the control and treatment regimens of 1.0, 1.5, 2.0, and 3.0 mM/mL BHT in BX and 0.5, 2.0, and 3.0 mM/mL in TEY and CEY extenders.

## Authors’ Contributions

AMK, NY, YR, MOA, and HW: participated in the study conception and design. AMK, HH, and KA: acquisition of data. AMK: analyzed and interpreted the data. AMK, and TKB: drafted the manuscript. All authors critically revised the manuscript for important intellectual content and approved the final manuscript.
